# Neuropsychological aspects of reversible cerebral vasoconstriction syndrome

**DOI:** 10.1590/1980-5764-DN-2023-0117

**Published:** 2024-07-08

**Authors:** Ícaro Araújo de Sousa, Analina de Freitas Azevedo, Arthur de Oliveira Veras, Marx Lima de Barros-Araújo, Elizeu Pereira dos Santos, Maria Andreia da Nóbrega Marques, Maria Paula Foss, Raimundo Pereira Silva-Néto, Irapuá Ferreira Ricarte, Octávio Marques Pontes-Neto

**Affiliations:** 1 Universidade de São Paulo, Faculdade de Medicina de Ribeirão Preto, Departamento de Neurociências e Ciências do Comportamento, Ribeirão Preto SP, Brazil.; 2 Universidade Federal do Piauí, Hospital Universitário, Departamento de Medicina Especializada, Teresina PI, Brazil.; 3 Universidade de São Paulo, Hospital das Clínicas, Instituto de Radiologia, São Paulo SP, Brazil.; 4 Universidade do Estado do Piauí, Departamento de Psicologia, Teresina PI, Brazil.; 5 Universidade de São Paulo, Faculdade de Filosofia, Ciências e Letras de Ribeirão Preto, Departamento de Psicologia, Ribeirão Preto SP, Brazil.; 6 Universidade Federal do Delta do Parnaíba, Departamento de Neurologia, Paraíba PI, Brazil.; 7 Universidade Federal de São Paulo Escola Paulista de Medicina, Departamento de Neurologia e Neurocirurgia, São Paulo SP, Brazil.

**Keywords:** Vasoconstriction, Cognitive Dysfunction, Neuropsychology, Ischemic Stroke, Vasoconstrição, Disfunção Cognitiva, Neuropsicologia, AVC Isquêmico

## Abstract

Despite the increasing knowledge in the past years, only minimal attention has been directed to the neuropsychological aspects and the prevalence of cognitive impairment associated with reversible cerebral vasoconstriction syndrome (RCVS).

**Objective:**

To describe the frequency and expand the understanding of cognitive dysfunction in RCVS.

**Methods:**

The neuropsychological evaluation was performed using a battery consisting of specific neuropsychological instruments that were administered to patients diagnosed with RCVS. A triage was conducted to exclude other potential causes of cognitive impairment. Performance on the tests was treated as a categorical variable, and a cutoff of −1.5 Z-score was adopted to indicate impaired performance.

**Results:**

Seven patients diagnosed with RCVS were evaluated, all of whom had a bachelor's degree and normal score in the Mini-Mental State Examination. The average time between diagnosis and neuropsychological evaluation was 1.8 years. Among the patients, 85.6% (n=6) exhibited performance below that of the normal population in at least two of the administered tests. Specifically, 71.4% (n=5) showed alterations in tests from the Psychological Battery for Attention Assessment, with impairment observed in concentrated (n=1), divided (n=3), or alternating (n=4) attention. Furthermore, 28.6% (n=2) demonstrated impairments in the Phonological Verbal Fluency Task, another 28.6% (n=2) exhibited difficulties copying elements of the Rey Complex Figure, and 14.3% (n=1) displayed lower performance in the Five-Digit test, all indicating executive dysfunction.

**Conclusion:**

This study provides evidence that cognitive impairment associated with RCVS is more prevalent than previously believed and has not received sufficient attention. Specifically, attention and executive functions are the cognitive domains most significantly impacted by RCVS.

## RESUMO.

Apesar do crescente conhecimento nos últimos anos, pouca atenção tem sido direcionada aos aspectos neuropsicológicos e à prevalência de declínio cognitivo associado à Síndrome de Vasoconstrição Cerebral Reversível (SVCR).

**Objetivo:**

Descrever a frequência e expandir o entendimento da disfunção cognitiva associada à SVCR.

**Métodos:**

A avaliação neuropsicológica foi realizada por meio de uma bateria composta de instrumentos neuropsicológicos específicos, que foram aplicados aos pacientes com SVCR. Uma triagem foi realizada para excluir outras potenciais causas de declínio cognitivo. O desempenho nos testes foi considerado como variável categórica, e o corte de −1,5 escore z foi adotado para indicar desempenho comprometido.

**Resultados:**

Sete pacientes com diagnóstico de SVCR foram avaliados, os quais tinham todos nível de ensino superior e pontuação normal no Miniexame do Estado Mental. A média de tempo entre o diagnóstico e a avaliação neuropsicológica foi de 1,8 ano. Dentre os pacientes, 85,6%(n=6) apresentaram desempenho comprometido em pelo menos dois dos testes aplicados. Especificamente, 71,4% (n=5) apresentaram alterações nos testes da Bateria Psicológica para Avaliação da Atenção, com comprometimento observado nas atenções concentrada (n=1), dividida (n=3), ou alternada (n=4). Além disso, 28,6% (n=2) demonstraram comprometimento na Fluência Verbal Fonológica, 28,6% (n=2) exibiram dificuldades na cópia da Figura Complexa de Rey e um paciente obteve desempenho alterado no Teste dos Cinco Dígitos, todos indicando disfunção executiva.

**Conclusão:**

Este estudo fornece evidência de que o declínio cognitivo associado à SVCR é mais prevalente do que se acreditava anteriormente. Especialmente, os domínios mais significativamente comprometidos foram a atenção e as funções executivas.

## INTRODUCTION

Reversible Cerebral Vasoconstriction Syndrome (RCVS) is characterized by diffuse segmental constriction of the cerebral arteries that spontaneously resolves itself within three months. Typically, it usually begins with acute and greatly intense and explosive headache, known as a thunderclap headache, that can be associated with focal neurological deficits related to swelling or cerebral ischemia^1^. Several names, such as "post-partum angiopathy", "drug-induced angiopathy", and Call-Fleming syndrome, have been given to this condition^2–4^. However, it was not until 2007 that Calabrese et al. proposed its current name and diagnostic criteria^5^.

The clinical manifestation usually follows an acute and self-limited course without new symptoms after one month^6^. However, complications and neurological comorbidities may occur during the acute phase of the illness, mainly a few days after the onset of symptoms^7^. Although most patients recover well and vascular abnormalities reverse spontaneously or after treatment with calcium-channel blockers, complications like extensive ischemic lesions, cerebral swelling, severe morbidity, or death may also occur^8–11^.

Yet none of these studies contain information about such cognitive status of the individuals, not even regarding those who had complications and presented persistent and significant neurologic deficits, after the angiopathy recovery. Thus, despite the exponential increase in knowledge about this disease in the past years, little attention has been directed to the neuropsychological aspects of RCVS.

This paucity of substantial evidence gave rise to several inquiries. Firstly, whether cognitive impairment is a rare outcome of the disease or a prevalent issue that has not been adequately studied yet. Secondly, whether there is a discernible pattern in cognitive occurrence among patients with RCVS. And thirdly, the central question is whether RCVS could lead to neuropsychological compromise independently of its complications. Hence, the present study aimed to amplify the understanding of cognitive dysfunctions in patients with RCVS.

## METHODS

### Participants

Patients diagnosed with RCVS were recruited from practices or public hospitals in the cities of Teresina (PI) and Ribeirão Preto (SP). Those who agreed to participate in the study signed the consent form and underwent a neuropsychological evaluation. This study protocol was reviewed and approved by the Research Ethics Committee of the Clinical Hospital of Ribeirão Preto School of Medicine (CEP/HCFMRP), with approval number 5.868.394.

### Inclusion criteria

- Age equal to or above 18 years.
- Definitive diagnosis of RCVS according to criteria defined by Calabrese et al. (2007). Angiography or indirect exam (Angio-MRI or CT) presenting multifocal segmental vasoconstrictions, acute severe headaches with or without neurological focal signs and complete reversibility of image findings after 12 weeks^5^.
- RCVS2 (diagnostic score) applied to all patients^12^.

### Exclusion criteria

- Evidence of subarachnoid hemorrhage (SAH) with aneurysmatic origin.
- Patients with cognitive impairment and another cause that justifies its deficits: degenerative diseases (Alzheimer's, typical or atypical parkinsonism, frontotemporal dementia), infectious diseases (neurosyphilis, human immunodeficiency virus [HIV] and acquired immunodeficiency syndrome [AIDS]), or metabolic diseases (severe hyperthyroidism, B12 deficiency, or alcohol-related dementia); suggestive findings in image exams that justify the presence of cognitive decline or vascular dementia in any of the subtypes — severe small vessel disease (radiologically defined as Fazekas scale grade II or III), strategic infarcts in cerebral areas or multiple cerebral infarcts — according to the revised criteria of Sachdev et al.^13^.
- Presence of confirmed vasculitis by serological or/and imaging tests.
- Patients with severe depression, defined by Beck depression inventory (BDI), with a score >19, without proper treatment^14^.

### Analyzed clinical and radiological variables

Epidemiological and clinical data were assessed, such as age, gender, and ethnicity; clinical data such as trigger factors or condition of the frame, associated complications, history of the thunderclap headaches at the beginning of the condition, functionality evaluation, previous depression or anxiety, previous migraine, neurological signs, and proposed therapeutic approach; and radiological, namely the presence of alterations in magnetic resonance imaging (MRI) and computed tomography (CT), presence of initial infarcts, presence of intraparenchymal hemorrhage (IPH), convexity subarachnoid hemorrhage (cSAH), vasogenic edema, condition recurrence, and involved arterial encephalic segments.

### Neuropsychological evaluation

Initially, patients underwent the Mini-Mental State Examination (MMSE) as a screening test. The neuropsychological evaluation included a battery of assessments formed by specific psychological and neuropsychological instruments recommended in the literature. The instruments and evaluated cognitive domains are listed below:

- Semantic Verbal Fluency (SVF) test-Animals — semantic fluency assessment^15^, also mental and cognitive flexibility related to the frontal lobe and executive functions^16^.
- Phonological Verbal Fluency (PVF) test — phonological fluency evaluation. It is sensible to evaluate the frontal lobe functions^17^.
- Boston Naming Test (BNT) — language assessment in naming aspects and semantic memory^18,19^.
- Five-Digit Test (FDT) — processing speed evaluation of attentiveness and executive functions^20^.
- Rey Complex Figure (RCF) — planning, visuo-constructive skills, and non-verbal cognitive function assessment^21^.
- Psychological Battery for Attention Assessment (BPA) — general attention capacity assessment and individually focused attention, divided attention, and alternated attention^22^.
- Beck Depression Inventory (BDI-II) — assessment of depression symptoms^14^.

The raw data of these instruments were transformed into Z-scores accordingly with normative values within their age reference and schooling groups. Then it was applied the Shapiro-Wilk test for a normality definition in the sample results. For the normal samples, it was selected a cutoff of −1.5 Z-score as a definition of performance below the normal population. For the samples that did not follow the regular distribution, it was considered an inferior performance result, those below the 10th percentile. Next, those variables were classified above or below the respective cutoff value for impairment. Accordingly, the patients’ performance were considered a categorical variable.

## RESULTS

### Epidemiological, clinical, and radiological data

Seven patients diagnosed with RCVS were analyzed, of whom 85.7% (n=6) were female. The patients’ average age was 47 standard deviation±13.2, and the most common ethnicity was Latin (42.8%), followed by white and black ethnicity (both 28.6%). Of the total, 71.4% of patients had a trigger condition, and all were taking antidepressants (80.0% serotonergic antidepressants), with only 28.6% using oral decongestants. Regarding the psychiatric disorder, 71.4% had anxiety (n=4) or depression (n=1). Only 28.5% of patients reported previous migraines.

All patients exhibited thunderclap headaches at the beginning of their condition and most (71.4%) had recurrence. Only one patient showed a focal neurological deficit in the condition manifestation, which was characterized by left temporal hemianopsia. One single patient exhibited a completely normal initial imaging exam (CT or MRI). Regarding the radiological findings, 28.5% (n=2) of patients displayed ischemia in initial exams, both at the right occipital location. cSAH in the initial exam was present in 57.1% (n=4) of patients. Only one had IPH in the left occipital location, associated with left parietal cSAH.

Each patient underwent digital subtraction angiography (DSA) or angioresonance (MRA) imaging as a brain vascular study. Only one did not exhibit initial alteration in the vessels study (MRA), but the presence of recurrent thunderclap headache associated with cSAH, and consequently an RCVS2 score of 7, sustained the diagnosed hypothesis. None of the patients presented internal carotid artery (ICA) intracranial involvement. The most frequently involved arteries were the anterior cerebral artery (ACA) and middle cerebral artery (MCA), representing 57.1%, followed by the posterior cerebral artery (PCA), with 42.9%. Apart from the patient who presented no alteration in the initial MRA, all the others exhibited vascular stenosis reversion. All patients displayed a good clinical outcome, demonstrated by modified ranking scale (mRS) scores of zero or one. Interestingly, one patient had a relapse in the condition approximately three years after the initial event.

All patients had an RCVS2 score ≥5. Concerning the appointed treatment, 71.4% (n=5) received nimodipine in the acute phase, one patient received verapamil, and another received other medications. Table 1 summarizes the main clinical/radiological characteristics of the patients.

**Table 1:** Epidemiological, clinical, and radiological data from the patients.

Patient	Clinical presentation at onset	Gender	Age	Race	Trigger associated condition	Prior comorbidities	Initial CT findings	Initial MRI findings	Angiography modality	Intracranial ICA involvement	Artery involved	Angiographic reversibility	mRS at discharge	Recurrence	RCVS2 score	Treatment
1	TCH + confusional state	Female	59	Black	Sertraline	Depression	Not performed	Convexity SAH	DSA	No	Bilateral ACA	Yes	0	No	10	Other medications
2	Recurrent TCH	Female	48	Hispanic/Latin	Absent	Absent	Convexity SAH	Convexity SAH, vasogenic edema	MRA	No	Bilateral MCA, bilateral PCA	No	0	No	7	Nimodipine
3	Recurrent TCH	Female	49	Black	Decongestant and fluoxetine	Anxiety	Left parieto-occipital IPH, convexity SAH	Not performed	DSA	No	Bilateral ACA	Yes	0	Yes	10	Nimodipine
4	TCH at wake up	Male	47	White	Absent	Absent	Not performed	Right occipital infarct	MRA	No	Right MCA (M2), right PCA (P2)	Yes	0	No	5	Nimodipine
5	Recurrent TCH	Female	30	Hispanic/Latin	Sertraline	Anxiety and migraine	Normal	Normal	DSA	No	Bilateral ACA. Bilateral MCA, bilateral PCA	Yes	0	No	9	Verapamil
6	Recurrent TCH + hemianopsia	Female	31	Hispanic/Latin	Trazodone	Anxiety and migraine	Right occipital infarct	Right occipital infarct	MRA	No	Right PCA (P1)	Yes	1	No	9	Nimodipine
7	Recurrent TCH + persistent headache with drowsiness	Female	65	White	Sertraline and isometheptene	Anxiety	Convexity SAH	Convexity SAH, vasogenic edema	DSA and MRA	No	Bilateral ACA, bilateral MCA	Yes	0	No	10	Nimodipine

Abbreviations: ACA, anterior cerebral artery; CT, computed tomography; ICA, internal carotid artery; IPH, intraparenchymal hemorrhage; MCA, middle cerebral artery; MRI, magnetic resonance imaging; mRS, modified ranking scale; PCA, posterior cerebral artery; SAH, subarachnoid hemorrhage; TCH, thunderclap headache; RCVS2, reversible cerebral vasoconstriction syndrome diagnostic score; DSA, digital subtraction angiography; MRA, magnetic resonance angiography.

### Neuropsychological evaluation

The transformation of raw data into Z-scores was made, presenting the achievement of each patient individually in each of the neuropsychological tests applied. All patients had bachelor's degrees and normal MMSE scores ≥28. The time average between RCVS diagnosis and neuropsychological evaluation was 1.8±1.05 years.

**Figure 1:**
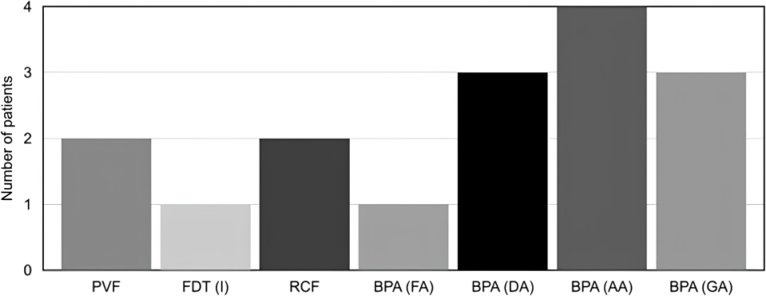
Number of patients for each compromised performance on neuropsychological tests.

Abbreviations: BPA, Psychological Battery for Attention Assessment; BPA (AA), BPA alternated attention; BPA (DA), BPA divided attention; BPA (FA), BPA focused attention; BPA (GA), BPA General Attention; FDT (I), Five-Digit test (inhibition); PVF, Phonological Verbal Fluency; RCF, Rey Complex Figures.

Among the assessed patients, 85.7% (n=6) exhibited an output below the normal population in at least two of the instruments in one cognitive domain or different ones. Specifically, 71.4% (n=5) showed alterations in tests from the BPA, with impairments observed in concentrated (14.2%), divided (42.3%), or alternating (57.1%) attention. Furthermore, 28.6% (n=2) demonstrated impairments in the PVF task, another 28.6% (n=2) exhibited difficulties copying elements of the RCF, and 14.3% (n=1) displayed lower performance in the FDT, all indicating executive dysfunction. The frequency of compromised performance on these neuropsychological instruments is presented in Figure 1. It can be remarked that none of the patients exhibited alterations in SVF, BNT, and RCF memory.

**Figure 2:**
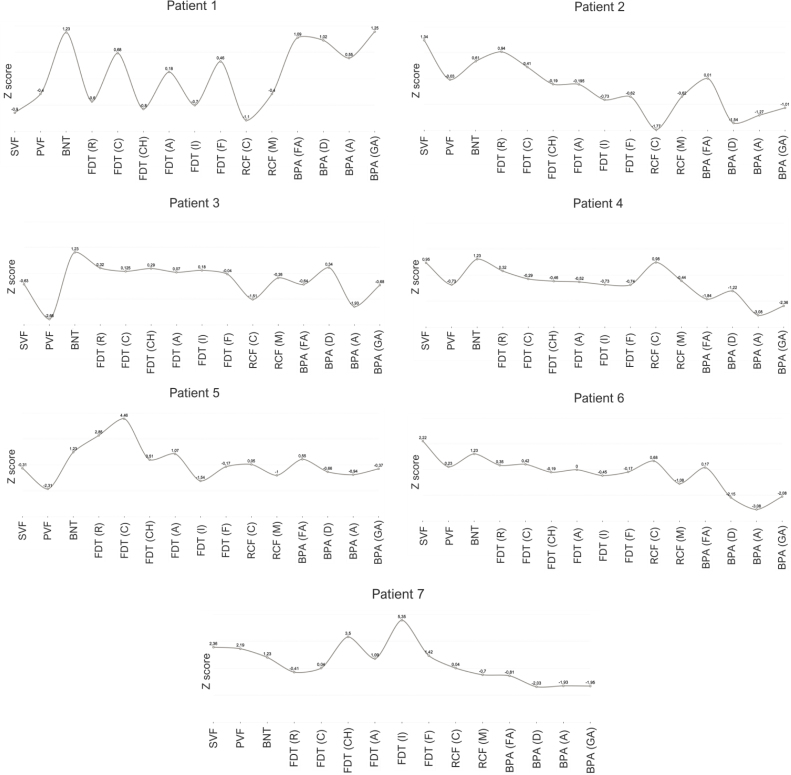
Individual performance of all patients on each neuropsychological test.

Abbreviations: SVF, Semantic Verbal Fluency; PVF, Phonological Verbal Fluency; BNT, Boston Naming Test; FDT, Five-Digit test; FDT (R), FDT reading; FDT (C), FDT counting; FDT (CH), FDT choice; FDT (A), FDT alternation; FDT (I), FDT inhibition; FDT (F), FDT flexibility; RCF, Rey Complex Figures; RCF (C), RCF copy; RCF (M), RCF memory; BPA, Psychological Battery for Attention Assessment; BPA (FA), BPA focused attention; BPA (D), BPA divided attention; BPA (A), BPA alternated attention; BPA (GA), BPA general attention.

When assessed individually, patients 4, 6, and 7 displayed inferior achievements only in BPA tests; patient 2 exhibited inferior achievement in BPA and RCF (copy); patient 5 presented inferior achievement in PVF, RCF (copy), and BPA. The graph in Figure 2 and Supplementary Materials (https://www.demneuropsy.com.br/wp-content/uploads/2024/05/DN-2023.0117-Supplementary-Materials.zip) represents the individualized achievement of each patient in the applied neuropsychological tests.

## DISCUSSION

We described a case series of patients diagnosed with RCVS who underwent neuropsychological evaluation. Most presented an inferior outcome in tests related to attention and executive functions in cognitive domains. To date, only one complete study had addressed this issue, which described a young patient who had multi-domain impairment initiated a few months after the syndrome. There is also a second case report, available only in abstract, which described dysfunction in visuospatial skills beyond what is expected due to the parenchymal lesion formed after resolution of vasoconstriction^23,24^.

Our population has characteristics similar to the cohorts described in the literature, especially relating to age average (47 vs 42 years); female prevalence; associated triggers (vasoconstrictor medication and serotonergic antidepressants); clinical presentation (thunderclap headaches); and prognosis, which is favorable in most cases^6^. Specifically, regarding associated comorbidities (psychiatric disorders), it could be inquired whether these would not alter the neuropsychological performance of the patients, once these disorders may be the cause of cognitive complaints (mostly depression) and since studies suggest that anxiety disorder alone does not cause cognitive decline^25^. However, of the five patients that took antidepressants, four were prescribed because of anxiety disorder and only one because of depression; besides, all were being treated for at least six months. The patient with depression presented with a BDI-II <19 and was receiving adequate treatment for more than six months.

Concerning radiological findings, most patients (n=4) had hemorrhagic complications, with three of them presenting cSAH alone and one presenting cSAH and IPH, in the locations occipito-parietal and parietal, respectively, both of small volume and occurring in the left hemisphere. Curiously, this patient was the only that presented no alteration on the neuropsychological tests. RCVS is commonly associated with hemorrhagic complications, the main one being cSAH followed by IPH, and is rarely associated with cerebral ischemia^26^. In our study, we conducted a comprehensive assessment of neuropsychological test results in patients with cSAH, revealing that three out of four exhibited cognitive performance alterations. This intriguing finding raises questions about the relationship between the location of cSAH and cognitive decline in individuals diagnosed with RCVS. While it is established that spontaneous subarachnoid hemorrhage is linked to cognitive decline, the evidence connecting cSAH to cognitive decline beyond cerebral amyloid angiopathy (CAA) remains limited^27^. However, it is crucial to emphasize that our findings do not definitively establish a causal link between cSAH and cognitive decline in RCVS patients. The extent to which cSAH may contribute to cognitive impairment in individuals with RCVS remains uncertain, and further research is needed to explore this potential association.

In the context of neuropsychological aspects, in our study, it is important to highlight that the relatively good performance observed in the MMSE within our sample may be due to its lower sensitivity in individuals with higher education levels, as well as its limited assessment of attention and executive functions^28,29^.

This emphasizes the necessity of including comprehensive neuropsychological assessments when evaluating patients with RCVS who report cognitive complaints. Additionally, we acknowledge the potential limitation of our study, which is the approximately 1.8-year gap between the onset of symptoms and the administration of neuropsychological assessments. This duration might have led to an underestimation of cognitive decline, particularly in light of findings from Perdices and Herkes^24^, suggesting that cognitive decline can manifest significantly within the first few months following the event. To definitively address this concern, a prospective study conducted immediately after the resolution of the RCVS condition would be required.

Nevertheless, the persistence of cognitive impairment beyond one year in our cohort raises the possibility that this clinical finding could be associated with persistent post-RCVS symptoms. Notably, previous research has demonstrated a 50% prevalence of headache persisting after three months of vasoconstriction resolution, with half of these patients requiring drug therapies for up to two years^30^. Given our study's primary goal of assessing chronic and potentially permanent cognitive decline associated with RCVS, we believe that our chosen timeframe was appropriate.

The main affected cognitive domain of patients was attention, evaluated by BPA tests (Figure 1). Attention is a cognitive domain that has a complex organization of multiple cerebral networks. Among them, is the dorsal attention network, associated with attention's external direction, related to the prefrontal cortex, ventral premotor, parietal, and temporal; the network "default mode", related to attention's internal direction, associated with the prefrontal ventromedial area, inferior parietal and posterior cingulate cortex; and the executive frontoparietal network that involves the lateral prefrontal cortex, anterior cingulate cortex, and inferior parietal^31,32^.

Next, the PVF and RCF tests exhibited a higher prevalence of abnormal performance (Figure 1); the fluency tests are essentially used for measuring executive function, and the PVF is sensible to assess frontal lobe functions, especially the left pre-frontal areas. However, as previously mentioned, RCF is a test that allows the assessment of several domains such as visuoconstructive functions, executive functions, and visual memory^16,20^. Particularly on the figure copying task, there is a specific recruitment of cognitive functions, like operative memory, inhibitory control, sustained attention, deep concentration, and information organization. These activities are intimately related to the frontal lobe functions and their connections, especially with the occipital and parietal, and they are related to executive functions^32,33^. Therefore, it is likely that the affected cognitive domains in these patients are the executive functions and the visuoconstructive ability with relative preservation of non-verbal memory. Yet there were no patients with alterations in evocation (second stage) of the RCF test.

Lastly, only one patient presented an abnormal performance at FDT, which assesses, among other abilities, the attention interference effect (Stroop effect), using conflicting information about numbers and quantities^20^. The compromise in inhibitory control for cognitive interference is common in executive dysfunction, which was also evident in the PVF alteration in this same patient (5). None of the patients presented abnormal performance in BNT and in the memory of RCF. Thus, in our population, cognitive functions of language and visual memory, at first, seemed preserved. Therefore, it is possible to conclude that the most affected cognitive domains in our population were attention and executive functions — both primarily located in the frontal lobe. The profile of neuropsychological impairment in this study differs from the report by Perdices and Herkes^24^, which besides presenting selective executive function deficits and a mild attentional deficit, revealed a severe decline in learning, delayed recall, and autobiographical memory. Possibly, we did not observe similar patterns in our patient group due to the absence of a more in-depth investigation into learning and memory.

Furthermore, despite the wide age range in our population (30–65 years), our average age closely aligns with that reported in the RCVS literature. We constructed the neuropsychological battery using tests with normative values adjusted for age and education, suggesting that age was not a decisive factor in cognitive decline. The elderly patient in our sample also performed satisfactorily in tests assessing various cognitive domains (excluding attention), suggesting the absence of other common causes of cognitive decline in individuals of this age group.

Regarding the physiopathological mechanism, current literature does not allow the establishment of neuropathological bases of how RCVS would cause cognitive impairment independently of its structural complications, but it is possible an association with cerebral vasoconstriction, as suggested by Perdices and Herkes^24^. In our population, as described, the main cerebral region related to inferior performance in the neuropsychological tests was the frontal lobe, and six patients presented involvement of arteries of anterior circulation (ACA, MCA, or both). However, since it regards complex cerebral functions, in which there is a wide neuron network involved, it is hard to establish a precise cause between the cognitive domains and impaired arteries.

If in the macroscopic field, the answer is uncertain, the molecular field can provide some clarification. It is known that the vasoconstriction action of catecholamines and endothelin-1 in the abrupt deregulation of cerebral vascular tonus is one of the component mechanisms of RCVS’ proposed physiopathology^34^. Besides, an experimental study with rats assessed the effect of intraventricular injection of endothelin-1 on the cognitive performance of these animals, and after seven days from the injection, there was an episodic memory decline. It also exhibited the reduction in CD31 expression in endothelial cells in the vessels surrounding the hippocampus and the "downregulation" of the cell signaling cascade proteins Akt1-mTOR, which led to the loss of activity-dependent protein translation, an essential factor in synaptic activity. Thus, this study shows that vasoconstriction may lead to cognitive function decline and poses protein translation as a key mechanism in synaptic plasticity. Curiously, this effect reverted after 30 days^35^. Still, in the molecular field, there has been a significant growth in the physiopathology of neurological diseases and microribonucleic acids (miRNAs). The miRNAs are small non-coding RNAs that regulate gene expression and are, presently, promising targets for interventions and mighty cell function regulators. Recently, some specific miRNAs were associated with hematoencephalic barrier dysfunction and cerebral vasomotor tonus loss in patients with RCVS^36^. Besides, evidence suggests a central role of miRNAs in mechanisms associated with the pathogenesis of vascular dementia, like in the regulating function of hematoencephalic barrier, apoptosis, oxidative stress, and neuroinflammation; and this has indicated these structures as potential biomarkers for cognitive decline^37^. Thus, miRNAs may be involved in the decline mechanisms associated with RCVS.

Our results should be interpreted in the context of the study design. First, the small number of patients hinders any analytical statistics, although the rarity of the disease makes studies with large populations difficult. Second, some cognitive domains were less represented by our neuropsychological test battery, like visuospatial functions and verbal memory, although they have been assessed indirectly. Further studies with a larger population and representativeness of the cognitive domains are necessary to clarify more about cognitive dysfunction in RCVS patients.

In conclusion, this study provides evidence that cognitive impairment associated with RCVS is more prevalent than previously believed and has not received sufficient attention. Importantly, attention and executive functions are the cognitive domains most significantly impacted by RCVS. These results underscore the need for increased recognition and consideration of cognitive deficits in patients with RCVS to ensure appropriate management and intervention strategies.
